# Targeting miR-9 in gastric cancer cells using locked nucleic acid oligonucleotides

**DOI:** 10.1186/s12867-018-0107-6

**Published:** 2018-06-07

**Authors:** Joana Filipa Lima, Joana Carvalho, Inês Pinto-Ribeiro, Carina Almeida, Jesper Wengel, Laura Cerqueira, Céu Figueiredo, Carla Oliveira, Nuno Filipe Azevedo

**Affiliations:** 1 Department of Chemical Engineering, LEPABE – Laboratory for Process Engineering, Environment, Biotechnology and Energy, Faculty of Engineering of the University of Porto, Rua Dr. Roberto Frias, 4200-465 Porto, Portugal; 2 Biomode, 2 S.A., Braga, Portugal; 3 i3S, Instituto de Investigação e Inovação em Saúde, Universidade do Porto, Porto, Portugal; 4 IPATIMUP, Institute of Molecular Pathology and Immunology of the University of Porto, Porto, Portugal; 5 FMUP, Faculty of Medicine of the University of Porto, Porto, Portugal; 6 National Institute for Agricultural and Veterinary Research (INIAV), Vairão, Vila do Conde Portugal; 7 Department of Physics, Chemistry and Pharmacy, Nucleic Acid Center, University of Southern Denmark, Odense, Denmark

**Keywords:** MicroRNA, miR-9, E-cadherin, LNA-AMOs, Locked nucleic acid, FISH

## Abstract

**Background:**

Gastric cancer is the third leading cause of cancer-related mortality worldwide. Recently, it has been demonstrated that gastric cancer cells display a specific miRNA expression profile, with increasing evidence of the role of miRNA-9 in this disease. miRNA-9 upregulation has been shown to influence the expression of E-cadherin-encoding gene, triggering cell motility and invasiveness.

**Results:**

In this study, we designed LNA anti-miRNA oligonucleotides with a complementary sequence to miRNA-9 and tested their properties to both detect and silence the target miRNA. We could identify and visualize the in vitro uptake of low-dosing LNA-based anti-miRNA oligonucleotides without any carrier or transfection agent, as early as 2 h after the addition of the oligonucleotide sequence to the culture medium. Furthermore, we were able to assess the silencing potential of miRNA-9, using different LNA anti-miRNA oligonucleotide designs, and to observe its subsequent effect on E-cadherin expression.

**Conclusions:**

The administration of anti-miRNA sequences even at low-doses, rapidly repressed the target miRNA, and influenced the expression of E-cadherin by significantly increasing its levels.

## Background

Gastric cancer (GC) is responsible for nearly 1 million deaths per year worldwide [1]. Like other cancers, the development of GC is a multistep process where the accumulation of genetic and epigenetic changes occurs [2]. E-cadherin inactivation is the most well-established defect in GC [3, 4]. In hereditary diffuse-type of GC and in sporadic GC with a diffuse component, the mechanisms that contribute to E-cadherin impairment are associated mostly with intragenic mutations, CDH1 locus deletions and promoter methylation, representing almost 50% of the cases. However, E-cadherin protein expression occurs in most GCs of both diffuse and intestinal type, so other mechanisms may occur to explain the overall defects on E-cadherin expression, such as miRNA posttranscriptional regulation.

MicroRNAs (miRNAs) are small, single-stranded and non-coding RNAs, with approximately 21 nucleotides that regulate gene expression at a posttranscriptional level [5–7]. They complementarily bind to the 3′UTR of their target mRNAs causing their degradation, translational repression, and/or deadenylation [8]. Upregulated miRNAs are often associated with protein downregulation being implicated in cancer and often displaying oncogenic activity [9–14]. Several studies have demonstrated that miR-9 is significantly upregulated in breast, liver, and colorectal cancer [15–20]. Our analysis of the miRNA profile of 37 cancer and 4 normal gastric tissue specimens, has also confirmed that miR-9 was overexpressed in gastric cancer tissues [4]. MiR-9 was shown to be responsible for the negative regulation of the *CDH1* gene, leading to tumor progression and invasiveness [21].

The targeting of aberrant miRNAs with antisense oligonucleotides (AMOs) has been put forward as a promising approach for post-transcriptional gene control in cancer therapy [22]. AMOs can act as competitive inhibitors of miRNAs, impairing their ability to interact with and repress cellular target mRNAs [23–31]. Locked Nucleic Acid (LNA) oligonucleotides are being used as AMOs due to their increased thermal stability and improved selective power with respect to their nucleic acid targets [23–27] to detect and to silence aberrant miRNAs in several human diseases, including cancer. Furthermore, they show low toxicity effects, being prone to be used in vivo for in situ localization of miRNAs in cells and tissues [27, 28].

**Fig. 1:**
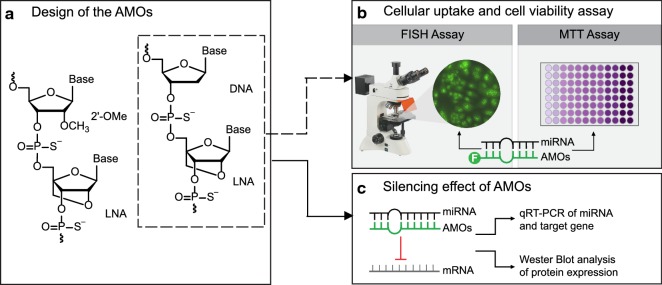
Schematic representation of the strategy applied in this work. **a** Design of the LNA-AMOs used in this manuscript. **b** Uptake and cytotoxic assessment of LNA-AMOs in cancer cell lines by fluorescence in situ hybridization (FISH) and MTT assay, respectively. **c** Silencing effect of the developed AMOs to the target miR-9 and its consequences on the expression of the E-cadherin by real-time PCR and Western blot analysis

In this work, we pursued an approach that enables miRNA antagonism in GC using nucleic acid mimics (Fig. 1). Our main goal was to design AMOs capable of detecting and silencing miR-9, with the aim of reestablishing E-cadherin expression, using the model of GC as a proof-of-concept. For that, two LNA-based AMOs with a complementary sequence to miR-9 were designed, their cellular uptake and cytotoxicity assessed, and their effects on the expression of miR-9 and of E-cadherin determined.

## Methods

### Cell lines and culture conditions

Human gastric cancer cell line MKN74 was purchased from the Japanese Collection of Research Bioresources Cell Bank (JCRB025, JCRB Cell Bank, Tokyo, Japan). HeLa (ATCC^®^ CCL-2™, derived from human cervix carcinoma) and human embryonic kidney 293 (HEK293, ATCC^®^ CRL-1573™) cell lines were purchased from the American Type Culture Collection (ATCC^®^, Rockville, MD, USA). MKN74 cells were maintained in RPMI medium 1640 Glutamax I (Gibco) supplemented with 10% (v/v) fetal bovine serum (FBS, HyClone, Thermo Fisher Scientific) and 1% (v/v) Penicillin/Streptomycin (Gibco). HeLa and HEK293 cells were cultured in Dulbecco’s Modified Eagle Medium (DMEM, Gibco, Thermo Fisher Scientific, Inc, UK) supplemented with 10% (v/v) fetal bovine serum (FBS, HyClone, Thermo Fisher Scientific, Inc, UK) and 1% (v/v) Penicillin/Streptomycin (Gibco). All cultured cells were maintained at 37 °C in a humidified 5% CO_2_ incubator and the medium was substituted every 2–3 days.

### LNA anti-miR design

The selection of the miR-9 target sequence was achieved by searching on the database TargetScan v7.1 [29–32]. Complementary LNA-based AMOs were designed taking into consideration optimal sequence length, specificity to the target, and theoretical melting temperature. Two LNA-based AMOs were synthesized with a phosphorothioate backbone (PS): an LNA/DNA anti-miR oligonucleotide sequence (AM_ LNA_PS) and another sequence harboring a 2′O-Methyl substitution (AM_LNA_2OMe_PS) (Fig. 1a). Additionally, two oligonucleotide sequences were used as a control: a scramble LNA sequence similar to the LNA/DNA AMO designed with 2 mismatches in the stretch targeting the seed region of mR-9 (SC_LNA_PS), and a commercial scramble sequence (SC_LNA_PS*). After the selection of the AMOs, a nucleotide alignment with Basic Local Alignment Search Tool (BLAST) was performed to ensure that the sequences were not targeting a similar region in another gene. The theoretical melting temperature (T_m_) of the AMO sequences was determined using the available online platforms, as an indicator of the hybridization temperature (T_H_). The T_m_ predictions for LNA/DNA sequences were evaluated using the Biophysics tool of IDT (available at http://biophysics.idtdna.com/). Predictions for the AM_LNA_2OMe_PS sequence were determined with the 2′OMeRNA/calculator (available at http://rnachemlab.ibch.poznan.pl/calculator1.php.

### LNA anti-miR synthesis

The oligonucleotide sequences used in this study were synthesized at the University of Southern Denmark as described elsewhere [33]. Briefly, the oligonucleotides were synthesized under anhydrous conditions using a standard automated nucleic acid synthesizer (Expedite 8909 instrument, PerSpective Biosystems Inc., MA, USA). LNA monomers were commercially available at Exiqon (Vedbaek, Denmark), and 2′-OMe were available at RiboTask ApS (Odense, Denmark). Synthesis was performed at the 0.2–1.0 μmol scale using a universal polystyrene-based support and the following conditions: trichloroacetic acid in CH_2_Cl_2_ (3:97) as detritylation reagent; 0.25 M 4,5-dicyanoimidazole (DCI) in CH_3_CN as activator; acetic anhydride in THF (9:91, v/v) as cap A solution; N-methylimidazole in THF (1:9, v/v) as cap B solution; and a thiolation solution containing 0.0225 M xanthan hydrate in pyridine/CH_3_CN (20:90, v/v). Coupling time was 4.6 min for both monomers. Fluorescein phosphoramidite (FAM, Glen Research, VA, USA) was added in anhydrous acetonitrile (0.1 M) and activated by tetrazole with a 20 min coupling time. The stepwise coupling yields (95–99% per step) were based on the absorbance of the dimethoxytrityl cations (DMT+) released after each coupling step. The cleavage from the support was carried out by using 98% aqueous methanol/ammonia solution 7 N in methanol (1:1), 2 h at room temperature (RT) followed by 32% aqueous ammonia solution, 12 h at 55 °C. The oligonucleotides were purified by reversed-phase HPLC (RP-HPLC) using a Waters 600 system equipped with an XBridge OST C18 (2.5 μm, 19 × 100 mm) column and an XBridge Prep C18 (5 μm, 10 × 10 mm) precolumn. After removal of the DMT-group, the oligonucleotides were characterized by ion-exchange HPLC (IE-HPLC) on a Dionex system HPLC (VWR) and by matrix-assisted laser desorption ionization time-of-flight mass spectrometry (MALDI-TOF) on a Microflex Maldi (Bruker instruments, Leipzig, Germany). The purified oligonucleotides were precipitated by acetone and their purity (> 90%) and compositions were verified by IE-HPLC and MALDI-TOF analysis, respectively.

### Cellular uptake

Cellular uptake of the FAM_AM_LNA_PS sequence was monitored by Fluorescence in situ hybridization (FISH) as described by Guimarães et al. [34] with modifications. MKN74 cells were cultured in a 24-well plate until reaching 70% of confluence. Then, the medium was discarded and the cells were fixed using a 4% paraformaldehyde solution, followed by a fixation with 50% ethanol. Each fixation was performed for 10 min at RT. After the fixation, it was added a hybridization solution containing the FAM_AM_LNA_PS at a final concentration of 5, 50 and 100 nM. Plates were then incubated at 37 °C for 2 h. Two types of hybridization solutions were tested: one containing 50% (v/v) formamide (Across Organic, NJ, USA), 10% (v/v) dextran sulphate (Fisher Scientific, MA, USA), 0.1% (v/v) Triton-X (Panreac, Barcelona, Spain), 5 mM of EDTA disodium salt 2-hydrate (Panreac), 50 mM Tris–HCl (Fisher Scientific NJ, USA), 10 mM NaCl (Panreac); and another containing urea (VWR BHD Prolabo, Haasrode, Belgium) with different concentrations (0.5 and 2 M) in 900 mM of NaCl (Panreac). After hybridization, the solution was discarded and it was added a pre-warmed washing solution containing 5 mM Tris Base (Fisher Scientific), 15 mM NaCl (Panreac) and 1% Triton X (Panreac) for 30 min at 37 °C. Coverslips were recovered from the plate and were mounted in a microscopic slide. For each experiment two negative controls were made: one using the same hybridization conditions, but without any oligonucleotide sequence in the solution, and another containing the scramble sequence (FAM_SC_LNA_PS).

For the assay using AMOs directly into the culture medium, after the seeding of the MKN74 cells, the medium was removed and the hybridization solution containing 0.5 M of urea and the FAM_AM_LNA_PS at a final concentration of 100 nM was added for 10 min. Afterwards, the medium was reestablished and the hybridization took place for an additional 2, 4, 8 and 48 h, at 37 °C in a 5% CO_2_ incubator. Then, cell slides were fixated and washed as described above. The microscopic slides were mounted using VECTASHIELD Antifade Mounting Medium with DAPI (Vector Laboratories, CA, USA). For image acquisition, an AxioImager Z1 (Carl Zeiss, Germany) epifluorescence microscope was used with a medium grid for optical sections of the fluorescent image. The FAM fluorochrome attached to the oligonucleotides was excited at 488 nm wavelength and the exposure time was fixed for all the preparations.

### Viability assay

The different cell lines were seeded at 1.6 × 10^5^ cells/well in 96-well plates and left 24 h to duplicate. Cells were then incubated with different concentrations (0.5–100 nM) of LNA-AMOs diluted in Phosphate Buffer Saline 1X (PBS), for 24 and 48 h. Cell viability was assessed by MTT (3-(4, 5-dimethylthiazolyl-2)-2, 5-diphenyltetrazolium bromide) assay using the CellTiter 96^®^ AQueous One Solution Cell Proliferation Assay (Promega Corporation, WI, USA) according to the manufacturer’s instructions. As a negative control, untreated cells exposed to the same conditions were used. Experiments were performed in triplicate.

### Total RNA extraction

Total RNA from each sample was extracted using the mirVana™ miRNA isolation kit (Invitrogen, Thermo Fisher Scientific, MA, USA), according to the manufacturer’s protocol. Shortly, the samples were lysed followed by an acid-phenol chloroform extraction. Total RNA was collected and small RNAs were purified by precipitation with ethanol. Small RNAs were immobilized on glass-fibre filters, washed and eluted with 30–60 μL of nuclease-free water. RNA yield and purity were determined by measuring the absorbance at 260 and 280 wavelengths in a NanoDrop^®^ Spectrophotometer (ND-1000; Fisher Scientific). Absorbance ratios (A260: A280) between 1.8 and 2.0 were considered optimal.

### Reverse transcription

The synthesis of single-stranded cDNA from total RNA samples for miRNA amplification was obtained using the TaqMan^®^ MicroRNA Reverse Transcription Kit (Applied Biosystems, CA, USA), according to the manufacturer’s protocol. In brief, each reaction consisted on 10 ng of small RNA, 0.15 μL of dNTP Mix with dTTP (100 mM total), 0.19 μL of RNase Inhibitor (20 U/μL), 1.5 μL of 10 × RT Buffer, 1 μL of Multiscribe™ RT enzyme (50 U/μL), 3 μL of 5 × RT Primers: hsa-*miR*-*9* (RT 583; Applied Biosystems) and RNU48 (RT 001006; Applied Biosystems) and nuclease-free water for a final volume of 15 μL. The reaction was performed at 16 °C for 30 min, 42 °C for 30 min followed by 85 °C for 5 min. The reverse transcriptase reaction for *CDH1* mRNA amplification was performed using 200 ng of RNA, 1 μL of random hexamer mix (MB129021, Nzytech, Lisbon, Portugal), 1 μL of dNTP Mix with dTTP (100 mM total), 1 μL of RNase Inhibitor (20 U/μL), 2 μL of 10 × RT Buffer, 1 μL of Multiscribe™ RT enzyme (50 U/μL) and nuclease-free water for a final volume of 20 μL. Each reaction was carried on a thermocycler according to the following program: 10 min at 70 °C, 2 min at 4 °C, and 1 h at 37 °C.

### Quantitative real-time PCR

qRT-PCR was performed for the relative quantification of miR-9 and of *CDH1* expression, in MKN74 cells after treatment with LNA-based AMOs in the conditions above. The qRT-PCR reaction for amplification of miR-9 was performed using the TaqMan MicroRNA Assays (TM 000583; Applied Biosystems), according to the manufacturer’s procedure. For the amplification of *CDH1* mRNA, 0.5 μl of TaqMan Real-Time probe was used in a PCR mixture containing 0.5 μl cDNA, 5.0 μl TaqMan 2X Universal PCR Master Mix No AmpErase^®^ UNG (Applied Biosystems). Reactions were incubated at 95 °C for 10 min followed by 40 cycles at 95 °C for 15 s and at 60 °C for 1 min and run on an Applied Biosystems 7500 Fast Real-Time PCR System. Each sample was amplified in triplicate. Relative miR-9 expression was normalized to levels of RNU48 (TM 001006; Applied Biosystems) endogenous control, and relative *CDH1* expression was normalized to levels of glyceraldehyde 3-phosphate dehydrogenase (Human GAPDH Endogenous Control FAM™ Dye/MGB Probe, Non-Primer Limited, Applied Biosystems). The Applied Biosystems 7500 Fast software was used to analyze the Ct values and the relative amount of miRNA and *CDH1* mRNA were determined using the 2^−ΔΔCt^ method.

### Western blot analysis

Western blot was performed to analyze the effect LNA-AMOs on the expression E-cadherin protein, under the same conditions stated above for the qRT-PCR analysis. MKN74 cells, treated or not with LNA-AMOs were washed three times with ice-cold PBS, and then disrupted using a lysing buffer, containing 1% Triton X-100 (v/v), 1% NP-40 (v/v, pH 7.4), supplemented with phosphatase inhibitor (1: 100) and protease inhibitor (1: 7) during 15 min. Samples were further centrifuged at 14,000 rpm for 20 min at 4 °C and the supernatants collected. 20 μg of total protein were mixed with laemmli 4x (Bio-rad, CA, USA), denatured at 95 °C for 5 min, and separated on a 10% polyacrylamide gel. Proteins were then transferred onto a nitrocellulose membrane and the membranes were blocked 30 min at RT with 5% dry milk in 0.5% Tween-PBS, followed by incubation at RT during 2 h with the antibodies in the following dilutions: a mouse monoclonal anti-α-E-Cadherin antibody (1: 1000) (Clone 36/E-Cadherin, BD Biosciences, CA, USA), and a mouse anti-α-GAPDH antibody (1: 30,000) (sc-47724, Santa Cruz Biotechnology, TX, USA). Blots were then washed 3 times with 0.5% PBS-Tween and incubated with a secondary anti-mouse antibody (1: 20,000) (sc-516102, Santa Cruz Biotechnology) for 2 h at RT.

### Image quantification

FISH images were analyzed using the ImageJ software (National Institutes of Health, MD, USA). Data was plotted as mean of arbitrary fluorescence units (AFU), representing the difference between the mean of fluorescence intensity of each sample and the background intensity values. Western Blot images were analyzed using the Quantity One^®^ 1-D software v4.6.6 (Bio-rad), and data was normalized to an endogenous control (GAPDH). The background values were obtained by measuring a blank region from each FISH and Western Blot image.

### Statistical analysis

All data are expressed as mean ± standard deviation. Each experiment was repeated at least 3 times. Statistical significance was determined using GraphPad^®^ Prism software v5.0.3. FISH results were analyzed using one-way analysis of variance (ANOVA) and Tukey’s multiple comparisons test. MTT, qRT.PCR, and Western Blot results were analyzed using two-way ANOVA and Tukey’s multiple comparisons test. Differences in data values were considered significant at *p* values lower than 0.05 (***p ≤ 0.001, **p ≤ 0.01, *p ≤ 0.05).

## Results

### Anti-miR oligonucleotides design and synthesis

Two LNA-based AMO sequences were designed with 16 nucleotides targeting specifically the miR-9 region responsible for *CDH1* dysregulation (AM_LNA_PS and AM_LNA_2OMe, Table 1). The AM_LNA_PS sequence is a DNA sequence with an LNA substitution in every 2 nucleotides, and the AM_LNA_2OMe, in addition to the LNA substitutions, contains 2′-OMe monomers along the sequence. AMOs harboring LNA modifications have been reported to show high thermal stability, resistance to nucleases and high efficiency on silencing miRNAs [23, 35, 36]. LNA substitutions combined with 2′-OMe modifications are known to further improve binding affinity when compared to oligonucleotides with only one type of substitution [37]. Additionally, two control sequences were synthesized, one designed to display two mismatches at the seed region (SC_LNA_PS), and the other is a scramble sequence with 19 nucleotides (SC_LNA_PS*) available commercially. Furthermore, the AM_LNA_PS and the scramble SC_LNA_PS were labeled with FAM (FAM_AM_LNA_PS and FAM_SC_LNA_PS, respectively, Table 1) for cellular uptake and viability assays. All sequences were designed with a PS backbone, which is reported to have increased anti-miRNA activity and enhanced cellular uptake compared to non-modified phosphodiester bonds (PO) [38].

**Table 1:** AMO designs used in this work and respective theoretical melting temperatures

Length (nt)	Name	Type	Sequence (5′-3′)	Predicted T_m_ (°C)
16	AM_ LNA_PS	LNA/DNA	GC+TAG+ATA+ACC+AAA+GA	57.92
16	AM_LNA_2OMe_PS	2′-OMe/LNA	gc+Uag+Aua+Acc+Aaa+Ga	77.60
16	SC_LNA_PS	LNA/DNA	GC+TAG+ATA+AGC+TAA+GA	51.95
19	SC_LNA_PS*	LNA/DNA	TAA+CAC+GTCT+ATAC+GCC+CA	61.03
16	FAM_AM_ LNA_PS	LNA/DNA	FAM_GC+TAG+ATA+ACC+AAA+GA	57.92
16	FAM_SC_LNA_PS	LNA/DNA	FAM_GC+TAG+ATA+AGC+TAA+GA	51.95

LNA nucleotide monomers are represented by the symbol “+” before the letter, 2′-OMe-RNA monomers in small caps letters, DNA nucleotides in capital letters, and FAM—fluorescein; the symbol asterisk refers to the scramble sequence available commercially

The substitution of DNA nucleotides by LNA monomers increases the predicted melting temperature of the sequences. Therefore, we determined the possible minimum length for AMOs that did not match additional off-targets and that kept a melting temperature value allowing the hybridization around 37 °C. Since there are no mathematical models to predict the T_H_ for these type of oligonucleotides, T_m_ values were predicted instead (Table 1), which are generally 15–30 °C higher than the T_H_ [39]. For that reason, the AMO sequences were designed in order to display a T_m_ ranging from 50 to 70 °C (Table 1).

### Screening of the cell lines for miR-9 content

**Fig. 2:**
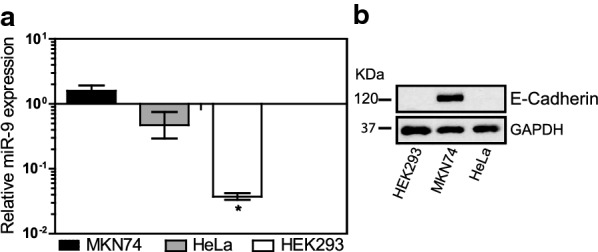
Expression profile of the cell lines used in this study. **a** Mir-9 expression assessed by qRT-PCR and **b** E-cadherin expression visualized by Western blot analysis

The cell lines used in this study were first tested by qRT-PCR to assess their miR-9 expression levels (Fig. 2a). Moreover, since it is expected that miR-9 antagonists induce re-expression of E-cadherin, the expression of E-cadherin expression level was also assessed by western blot (Fig. 2b). With exception of the MTT assay, the following experiments were performed using MKN74 cells, since this cell line expresses both miR-9 and E-cadherin (Fig. 2). This cell line while not presenting a high content on miR-9 expression when compared to other gastric cancer cell models [40], it presents an unaltered E-cadherin expression, and for that reason it was chosen for this study. In the cell viability studies, all cell lines were used, regardless of miR-9 and E-cadherin expression, to test the cytotoxic effect of AMOs in different tumor cell-types.

### Cellular uptake of AMOs and their effect on cell viability

After designing the AMOs, we investigated their ability to enter cancer cells. For that, the cellular uptake of the FAM_AM_LNA_PS oligonucleotide was monitored by FISH using the miR-9-positive MKN74 cell line.

The first FISH protocol tested comprehended a hybridization solution containing, among other components, 30% formamide as a destabilizing agent. However, it was not possible to visualize a fluorescence signal, even when protocol adjustments, such as hybridization, washing, and fixation time were made (data not shown). As an alternative strategy to formamide, a hybridization buffer containing urea was used for the subsequent FISH assays.

**Fig. 3:**
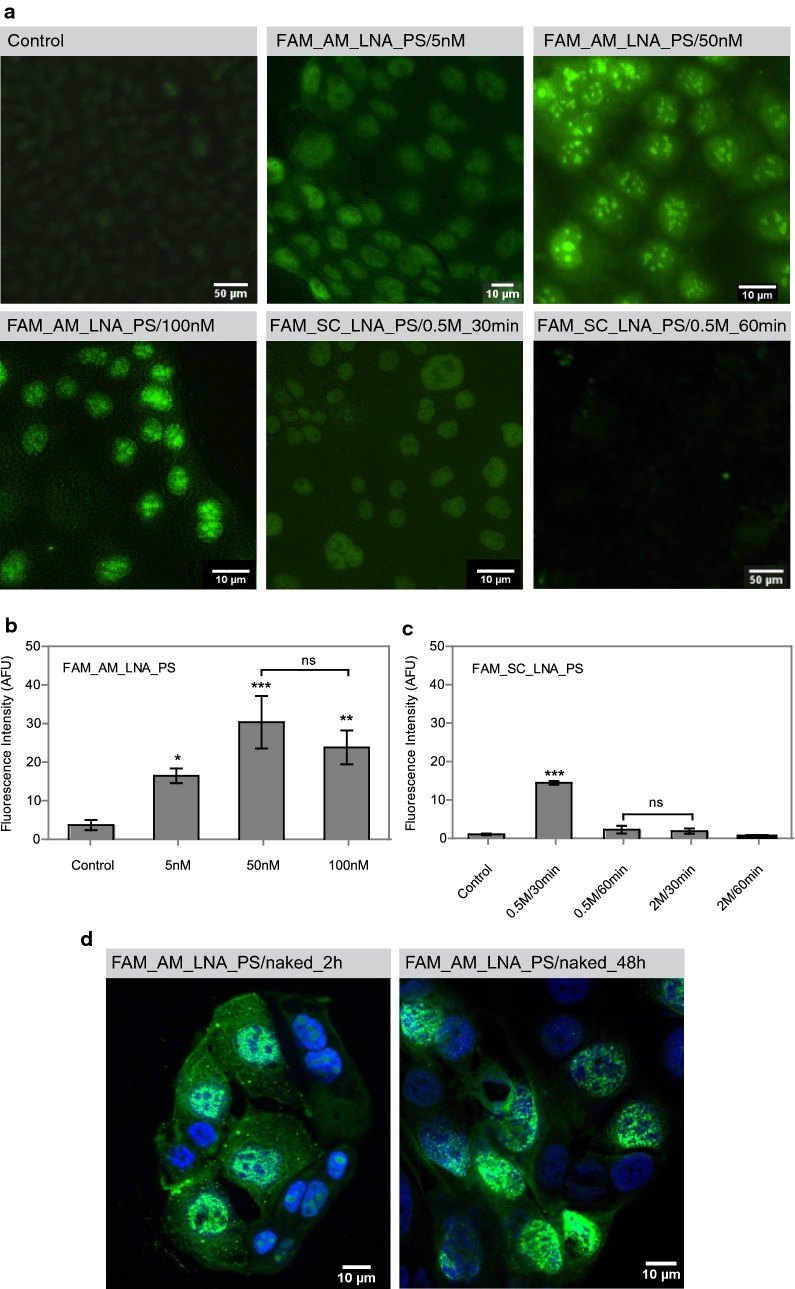
Cellular uptake assay of the labeled AMOs using MKN74 cells. **a** Representative epifluorescence microscopy images obtained with FAM_AM_LNA_PS at different concentrations (FAM_AM_LNA_PS/5 nM,/50 nM and/100 nM) in a hybridization solution containing 0.5 M of urea. The hybridization step was performed at 37 °C for 2 h, followed by 30 min of washing. As negative controls, cells without any sequence (control) or incubated with FAM_SC_LNA_PS were used. The FAM_SC_LNA_PS/0.5M_30 min, and FAM_SC_LNA_PS/0.5M_60 min corresponds to the FISH assay using the FAM_SC_LNA_PS hybridized for 2 h using a solution containing 0.5 M of urea, followed by a washing step of 30 and 60 min, respectively. All images were acquired using an ×200 magnification. **b** Average fluorescence intensity for the FAM_AM_LNA_PS at 5, 50, and 100 nM; and **c** for the FAM_SC_LNA_PS using different concentrations of urea in the hybridization solution and washing times. Fluorescent signal intensity is expressed in arbitrary fluorescent units (AFU); *ns*—not significantly different from control *(p *> 0.05). **d** Cellular uptake assay using the FAM_AM_LNA_PS at 100 nM (FAM_AM_LNA_PS/naked) applied directly into the medium after 2 and 48 h of hybridization. DAPI was used to stain the nucleus. The image was acquired using an ×400 magnification

Using urea in the hybridization solution, fluorescence signals were detected for all tested concentrations (FAM_AM_LNA_PS at 5, 50 and 100 nM, Fig. 3a), with significantly higher intensities at concentrations of 50 nM and 100 nM (Fig. 3b). As expected, the negative control, i.e. cells without treatment with AMOs, did not display any fluorescent signal (control, Fig. 3a).

The FAM_SC_LNA_PS was used as another negative control at a 50 nM concentration. This concentration was selected based on the results with FAM_AM_LNA_PS, which showed no statistically significant differences between concentrations of 50 nM and 100 nM (Fig. 3b). Using the same FISH settings, the FAM_SC_LNA_PS showed a fluorescence signal in the same range of the lowest FAM_AM_LNA_PS concentration (5 nM, Fig. 3b and 3c). In order to improve AMOs specificity and to eliminate off-target signal, the FISH protocol was adjusted for a longer washing time and and/or higher urea concentration. An extended washing step (Fig. 3a, SC_LNA_PS/0.5M_60 min) was sufficient to increase the hybridization stringency and reduce the FAM_SC_LNA_PS signal (Fig. 3c).

To further investigate the cellular uptake of AMOs in a setting without carriers or transfection agents, the FAM_AM_LNA_PS and correspondent scramble sequence were added directly in the culture medium. Remarkably, a positive fluorescence signal was observed as early as 2 h after the AMOs addition (FAM_AM_LNA_PS/naked_2 h, Fig. 3d). Furthermore, the fluorescence signal was detected at all time-points, even after 48 h of incubation (FAM_AM_LNA_PS/naked_48 h, Fig. 3d). Although the signal was not observed transversely in all the cells, it was still possible to detect fluorescence and to demonstrate that LNA-based AMOs are able to endure in the medium for long periods. Moreover, the LNA-AMOs were able to bind the target miR-9 in the absence of denaturant agents or carriers.

### AMOs effect on cell-lines viability

**Fig. 4:**
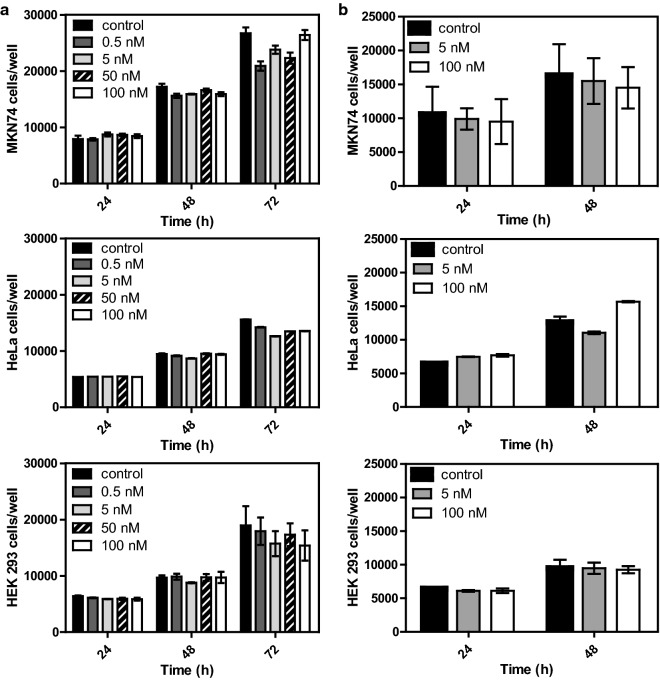
MTT viability assay results for the different cell lines MKN74, HeLa and HEK293. **a** Cell lines treated with FAM_AM_LNA_PS and with **b** FAM_SC_LNA_PS. The control is related to the cells without any AMOs treatment

After confirming the cellular uptake of the LNA-based AMOs, the effect of AMOs in cell viability was assessed in a panel of cell lines. It was decided to use the sequences labeled with FAM to further evaluate the effect of the fluorochrome in the cell viability. MTT assay results showed that, up to a concentration of 100 nM, FAM_AM_LNA_PS did not significantly reduce cell viability in any of the cell line models used (Fig. 4a). These results suggest that LNA-based AMOs are not cytotoxic.

The MTT test was also performed using the FAM_SC_LNA_PS sequence at 5 nM and 100 nM for 24 and 48 h. Since the FAM_AM_LNA_PS did not show significant cytotoxic effect in vitro, we tested the effect of the scramble sequence on cell viability only for the minimum and maximum values of concentration used for the FAM_AM_LNA_PS. The FAM_SC_LNA_PS also did not significantly compromise cell viability (Fig. 3b), reinforcing that LNA-based AMOs are not cytotoxic. Moreover, since both sequences were labeled with FAM and both did not significantly reduced cell viability, this labelling was also considered not cytotoxic.

### Effects of AMOs in the expression of miR-9 and of E-cadherin

The AM_LNA_PS and another chemically-modified oligonucleotide (Table 1) were used for evaluating the effectiveness of AMOs on the silencing of miR-9.

The SC_LNA_PS sequence had no effect in the expression of miR-9, presenting similar results to the SC_LNA_PS* sequence (data not shown), and was thus suitable to function as a negative control.

**Fig. 5:**
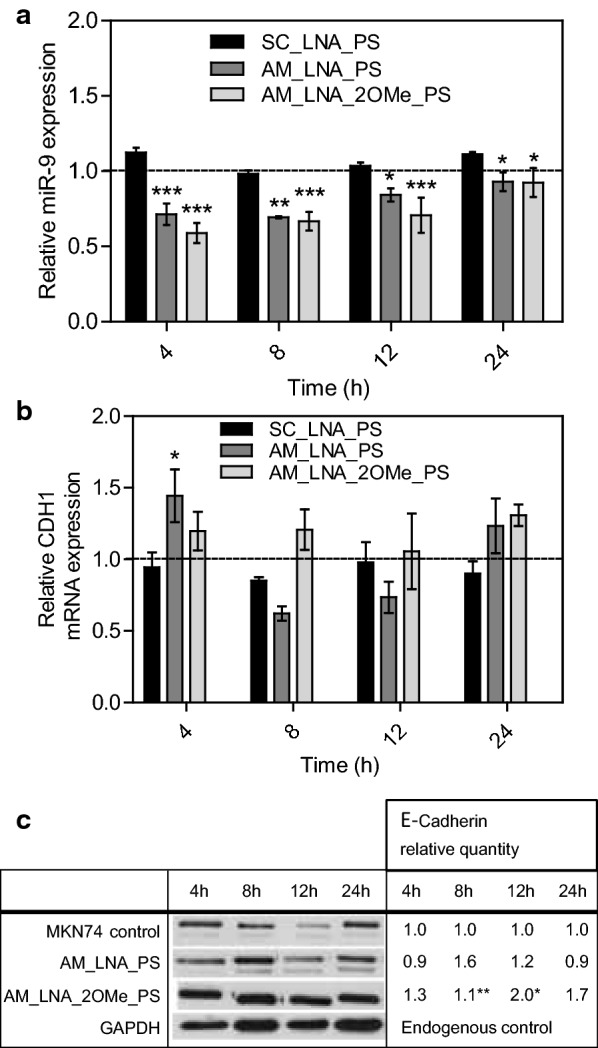
Silencing effect of the AMOs in MKN74 cells. Relative expression levels (measured by qRT-PCR) of **a** miR-9 and **b** *CDH1* mRNA, after 4, 8, 12 and 24 h treatment with AM_LNA_PS, AM_LNA_2OMe_PS, and SC_LNA_PS. **c** Western blot analysis of the relative variation of E-cadherin expression in MKN74 cells after treatment with LNA-anti-miR oligonucleotides and the control, MKN74 cells without any treatment, at the same time-points used for qRT-PCR. All the qRT-PCR and western blot results were normalized against the MKN74 control and the levels of the respective endogenous control (RNU48 for miR-9 and GAPDH for *CDH1* gene quantified by qRT-PCR and E-cadherin protein expression by western blot)

The AM_LNA_PS significantly decreased the miR-9 expression in the first 8 h of incubation. After that time-point, cells started to recover miR-9 content until basal levels (Fig. 5a). On the other hand, the *CDH1* mRNA twofold variation showed a significant increase after 4 h of incubation, probably as a response to the decrease on miR-9 expression at the same time-point. After that, a decrease on *CDH1* expression is observed, before its stabilization to basal levels (Fig. 5b).

The AM_LNA_2OMe_PS influenced the expression of miR-9 during a longer period. A decrease of miR-9 expression levels was visible after 4 h, which was maintained up to 12 h of incubation (Fig. 5a). After that point, there was an increase of miR-9 expression levels until the basal miRNA level. In terms of *CDH1* mRNA expression, with the exception of the 4 h time-point, there is no significant increase on *CDH1* mRNA relative expression, despite the significant decrease in miR-9 expression (Fig. 5b).

When analyzing the results by western blot (Fig. 5c), in all the situations it is evident an effect on the E-cadherin expression in comparison with the control. In the cells treated with the AM_LNA_PS, there is a slight increase in E-cadherin expression after 8 h of incubation, which then remains stable in the following time points. These results are in accordance with the qRT-PCR data, with a small delay between the *CDH1* mRNA relative content and the consequent effect on the protein expression (Fig. 5b, c). In cells treated with the AM_LNA_2OMe_PS, the levels of E-cadherin increased at all time points, especially at 8 and 12 h, where a significant increase of protein expression was observed (Fig. 5c).

## Discussion

Overexpressed miRNAs are often associated with the downregulation of many tumor suppressing proteins, and thus being implicated in the pathogenesis of several human diseases, including cancer. In GC, miR-9 has been reported to be mainly downregulated, functioning as a tumor suppressor by targeting *RAB34* (encoding the Ras-related protein RAB-34) and *NFKB1* (encoding the NF-kappa-B transcription factor) oncogenes [41, 42]. However, miR-9 has also been shown to be upregulated in GC, where its overexpression leads to the downregulation of the *CDX2* gene, which encodes a human caudal-type homeobox protein, CDX-2, promoting cell proliferation and invasion [40].

In humans, miR-9 can be transcribed from three independent genomic loci, resulting in three pri-miRNAs (miR-9-1, miR-9-2 and miR-9-3). Those three pri-miRNAs can be further processed to give rise to two functional mature miRNAs: miR-9-3p and miR-9-5p, being the later the most investigated mature miR-9. The expression levels of miR-9-5p are highly variable across cancers, being either upregulated or downregulated. Also, it has been shown that the same miRNA gene can behave as oncogene or tumor suppressor gene in the same type of cancer, depending on the type of alteration, cell type or transcriptional/posttranscriptional events. Due to their small size, point mutations are less probable to occur, and the main mechanisms for miRNA inactivation or activation seem to be correlated with homozygous deletions, the combination mutation plus promoter hypermethylation (in case of miRNA inactivation/underexpression) or gene amplification (in case of miRNA activation/overexpression). Due to the accumulating evidence of miR-9 overexpression in a panel of 37 GC specimens, and that its overexpression might influence the expression of E-cadherin, the AMOs developed in here were designed to specifically bind to the miR-9-5p region that targets the *CDH1* 3′UTR mRNA.

Before moving to a treatment based approach, the ability of AMOs to provide a fluorescence signal that can be used as a diagnostic marker was evaluated. Herein, after optimization of a standard FISH protocol, it was possible to detect a specific FISH signal using the FAM_LNA_PS at 50 nM in a hybridization buffer containing urea instead of formamide. Formamide has been extensively used for over 30 years as an RNA stabilizer to reduce incubation temperature and/or heterologous background hybridization that often occurs with RNA probes [43]. Even though its advantageous hybridization features, formamide is very toxic and one of the most hazardous chemicals identified by regulatory agencies [44]. Probably due to the high toxicity profile, the in vitro models could not endure the FISH treatment, leading to an unsuccessful hybridization of the AMOs used in this study. Several studies report the use of urea in hybridization solution as an alternative to formamide [45–47]. Hybridization buffers containing urea are less toxic and enclose far less chemical compounds comparing to the ones with formamide [48]. The urea-based hybridization solution has provided a strong and specific fluorescence signal, especially when combined with longer washing periods (60 min).

Additionally, the ability of AMOs to diffuse through the cell membrane and bind to the miRNA in the absent of a carrier or a denaturant solution was evaluated by adding AMO directly to the culture media. Fluorescence signal was observed as soon as after 2 h of incubation with the AM_LNA_PS sequence. PS-oligonucleotides targeting miRNAs have been shown to be rapidly internalized by cells, both in vitro and in vivo, in the absence of any carriers, and to interfere with miRNA activity in a process called Gymnosis [49, 50]. Gymnotic studies, however, tend to use higher concentrations of AMOs for longer periods of time [49, 51]. From our study, LNA-based AMOs are able to enter the cells without any carriers or transfection agents and to specifically bind and inhibit miR-9 expression. Further tests on cellular viability have shown that AMOs were not toxic to any of the three cell-lines tested.

Moreover, the LNA-AMOs tested here were able to repress the miR-9 expression at early time points and at a low concentration. There is evidence that the PS-AMOs internalization occurs via the endosomal pathway, through which the oligonucleotides might meet the miRNA within, blocking its action [51]. This silencing mechanism is still not yet validated for all cell types and miRNAs. Nevertheless, it seems that the PS-AMOs do not necessarily need to be released from endosomes to act upon their target miRNA. This mechanism might explain the fact that the AMOs tested in this study were able to silence the target miRNA in such a short period of time.

LNA-modified AMOs have been reported to specifically inhibit miRNAs leading to the up-regulation of the target proteins [25, 26, 35, 37]. The 2′OMe modification associated with LNA monomers is known to display increased potency and stability on silencing aberrant miRNAs [52]. Accordingly, the AM_LNA_2OMe_PS used herein showed a more extensive effect on repressing miR-9 levels, when compared with the other design.

Overall, for the AMOs tested, it was not visible an inversely proportional variation on *CDH1* mRNA relative expression level as a possible consequence of the miR-9 silencing. The slight increase in the *CDH1* mRNA expression level along time could be explained by the fact that we only tested a low-dose of AMOs and in a relatively short period of time. Also, the AMOs tested, despite designed to target specifically the miR-9 loci believed to promote E-cadherin impairment, they might target other miR-9 precursors. As a consequence, the decrease of miR-9 content might be more extensive than the effect on the *CDH1* mRNA expression level. Another factor to have in consideration is that at this early stage we believe the AMOs only meet the miRNA in the endosomes, and probably due to this do not reach all the available miR-9 available. Therefore, the AMOs presented in here might present higher potency on silencing the target miRNA in a setting using a broader delivery system, higher AMO concentrations and/or longer incubation periods.

Regarding the protein expression, it was observed a delay between the effect on the relative content of *CDH1* mRNA and the consequent impact on the protein expression, due to the normal time-gap between mRNA re-expression and initiation of the protein translation. The AMOs tested have roughly followed the same pattern between the results obtained by mRNA qRT-PCR and protein quantification. For the AM_LNA_PS, after 8 h of incubation there is a noteworthy decrease on *CDH1* mRNA expression. The reason for this result is not very clear. We hypothesize that due to the tight regulation system of the MKN74 cells, as the cellular machinery is trying to regain miR-9 function, probably by overproducing the miRNA to compensate the effect of the anti-miR sequence, it might have destabilized the expression of *CDH1* mRNA.

The therapeutics involving AMOs is a promising approach towards an effective treatment of several human pathologies caused by the dysregulation of miRNAs. Currently, three LNA-AMO-based drugs are undergoing clinical trials: one called Miravirsen developed by Santaris Pharma to control the activity of miR-122 associated with Hepatitis C virus (HCV) infection [53]; and two others from miRagen Therapeutics to inhibit miR-155 (MRG-106) and miR-92a (MRG-110, S95010) involved respectively, in blood cancers and in acute cardiovascular diseases. Despite of the promising results, no therapeutic strategy has been yet fully developed and validated. The AMO technology can also be further associated with emerging methodologies for real-time detection of miRNAs in humans. In situ detection of miRNA accumulation in tumor biopsies may provide a highly valuable approach for prognostic and diagnostic evaluation in a clinical setting. In addition, little is still known on how the biological systems regulate miRNAs expression in the different human diseases [54]. The double role of miRNAs requires a more intense study in order to develop reliable, specific and safe technologies to fully control miRNA expression. AMOs are indeed a great tool, not only for the detection and therapeutics, but also for applications that help to unravel the mechanisms behind miRNA biogenesis and function.

## Conclusions

A quite straightforward approach to target aberrant miRNAs is to develop molecules that specifically binds to the target miRNA, blocking its function. In this case, by targeting overexpressed miR-9 in GC cell lines using AMOs, it was expected to promote the normal function of *CDH1*-encoding mRNA and this way reestablish the expression of E-cadherin. In addition to the therapeutic approach, this study also aimed to explore the potential of AMOs combined with the FISH technology to effectively detect in vitro overexpressed miRNAs and their relevance to function as miRNA markers involved in different human diseases.

In here, we have demonstrated the potential of naked LNA-based AMOs to enter cells using low AMO concentrations in a relatively short period of action. Moreover, all the sequences were able to silence the expression of upregulated miR-9 using a cancer cell model. Even though the results on the E-cadherin expression were not so straightforward, there is a slight increase on protein expression. Overall, the LNA_2OMe_PS oligonucleotide sequence seems to be the design with more promising results on the silencing of miR-9.

Furthermore, the designed AMOs might present higher potency on silencing the target miRNA in a setting using a broader delivery system, higher AMOs concentration and/or longer incubation periods. Also, these sequences could be further improved and modified to target other overexpressed miRNAs within GC and in other human disorders.

## Abbreviations

CG: Gastric cancer
LNA: locked nucleic acid
PS: phosphorothioate
PO: phosphodiester
AMO: anti-miRNA oligonucleotide
miRNAs: microRNA
AM: anti-miR
SC: scramble
2’-OMe: 2′O-methyl
FAM: fluorescein phosphoramidite
FISH: fluorescence in situ hybridization
T_m_: melting temperature
T_H_: hybridization temperature
RT: room temperature
PBS: phosphate buffer saline
*CDH1*: E-cadherin encoding gene
*RAB34*: Ras-related protein RAB-34 encoding gene
*NFKB1*: nuclear factor NF-kappa-B p105 subunit encoding gene
*CDX2*: homeobox protein CDX-2 encoding gene
*MYC*: myc proto-oncogene protein encoding gene
